# A Nanocrystalline Fe_2_O_3_ Film Anode Prepared by Pulsed Laser Deposition for Lithium-Ion Batteries

**DOI:** 10.1186/s11671-018-2475-8

**Published:** 2018-02-23

**Authors:** Xiaoling Teng, Youzhi Qin, Xia Wang, Hongsen Li, Xiantao Shang, Shuting Fan, Qiang Li, Jie Xu, Derang Cao, Shandong Li

**Affiliations:** College of Physics Science, Qingdao University, No.308 Ningxia Road, Qingdao, 266071 China

**Keywords:** Lithium-ion batteries, Nanocrystalline Fe_2_O_3_, Anode material

## Abstract

Nanocrystalline Fe_2_O_3_ thin films are deposited directly on the conduct substrates by pulsed laser deposition as anode materials for lithium-ion batteries. We demonstrate the well-designed Fe_2_O_3_ film electrodes are capable of excellent high-rate performance (510 mAh g^− 1^ at high current density of 15,000 mA g^− 1^) and superior cycling stability (905 mAh g^− 1^ at 100 mA g^− 1^ after 200 cycles), which are among the best reported state-of-the-art Fe_2_O_3_ anode materials. The outstanding lithium storage performances of the as-synthesized nanocrystalline Fe_2_O_3_ film are attributed to the advanced nanostructured architecture, which not only provides fast kinetics by the shortened lithium-ion diffusion lengths but also prolongs cycling life by preventing nanosized Fe_2_O_3_ particle agglomeration. The electrochemical performance results suggest that this novel Fe_2_O_3_ thin film is a promising anode material for all-solid-state thin film batteries.

## Background

With the ever-increasing applications of lithium-ion batteries (LIBs) in portable electronics and electric vehicles, there has been extensive research on developing advanced electrode materials with higher energy and power densities [1–7]. Since the first report on reversible lithium storage in transition metal oxides (TMOs) by Poizot et al. [8], TMOs (Co_3_O_4_ [9, 10], NiO [11, 12], Fe_2_O_3_ [13–15], and CuO [16, 17]) have been widely explored as anode materials due to their higher theoretical specific capacity and better safety in comparison with traditional carbon anode materials. Among all these TMOs, Fe_2_O_3_ received much attention in recent years due to its high theoretical specific capacity (~ 1005 mAh g^− 1^), low cost, abundant resources, and environmental benignity. However, like other TMOs, the huge volume variations associated with Li-ion insertion/extraction often leads to the pulverization and subsequent falling off of the active materials from the electrode, which results in a significant capacity fade, poor cycling stability, and poor rate capability. To circumvent these problems, many nanostructures of Fe_2_O_3_ have been synthesized for lithium-ion batteries, such as nanorods [18, 19], nanoflakes [20, 21], hollow sphere [22–24], core-shell arrays [25], and micro-flowers [26].

Besides all the above nanostructures, nanocrystalline thin film anodes (NiO [27], MnO [28], Cr_2_O_3_ [29], CoFe_2_O_4_ [30], Si [31], and Ni_2_N [32]) deposited directly on conducting substrates by pulsed laser deposition or sputtering can also exhibit an excellent electrochemical performance due to the enhanced electrical contact between the substrates and active materials, the shortened diffusion lengths for lithium-ion, and the structure stability. What is more important is that thin films of TMOs have potential applications in all-solid-state microbatteries as self-supported electrodes [33, 34]. The TMOs’ films can replace the lithium film anode which limits the integration of microbatteries with circuits due to the low melting point and strong reactivity with moisture and oxygen. However, up to now, there have been few reports on the Fe_2_O_3_ film anodes deposited by pulsed laser deposition or sputtering, and the reported specific capacities were much lower than the theoretical specific capacity of Fe_2_O_3_ [35, 36].

In this work, we prepared nanocrystalline Fe_2_O_3_ films by pulsed laser deposition (PLD) as an anode material for lithium-ion batteries. The Fe_2_O_3_ thin film anodes with average grain size of several tens of nanometers showed high reversible capacity of 905 mAh g^− 1^ at 100 mA g^− 1^ and high rate capacity of 510 mAh g^− 1^ at 15000 mA g^− 1^. The remarkable electrochemical performance demonstrates that nanocystalline Fe_2_O_3_ thin film has potential applications in high performance LIBs, especially all-solid-state thin film batteries.

## Experimental

### Synthesis of Nanocrystalline Fe_2_O_3_ Films

The films of Fe_2_O_3_ were deposited directly on copper foils or stainless steels by a PLD technique in oxygen ambient. A KrF excimer laser with a wavelength of 248 nm was focused on the rotatable target of metal Fe. The repetition rate was 5 Hz, and the laser energy was 500 mJ. The distance between the target and the substrate was 40 mm. In order to get nanocrystalline Fe_2_O_3_ films, we grew samples at room temperature under oxygen pressure of 0.3 Pa on both copper foil and stainless steels. They showed the same electrochemical performance. The thickness of the nanocomposite film is approximately 200 nm as determined by atomic force microscope (AFM, Park systems XE7). The mass of 0.121 mg was obtained by measuring the difference of substrate before and after deposition via electrobalance (METTLER TOLEDO).

### Material Characterization

The crystalline phase of the Fe_2_O_3_ film was characterized by X-ray diffraction (XRD) on a Rigaku D/Max diffractometer with filtered Cu Kα radiation (*λ* = 1.5406 Å) at a voltage of 40 kV and a current of 40 mA. High-resolution transmission electron microscopy (TEM) and selected area electron diffraction (SEAD) were carried out by a JEOL 100CX instrument. For the TEM measurement, the Fe_2_O_3_ film grown on NaCl substrate was put into water to dissolve the NaCl. After that, the suspension was dropped onto a holey carbon grid and dried. The morphology of the samples were observed by scanning electron microscopy (SEM) using a SU8010. X-ray photoelectron spectroscopy (XPS) measurement was performed on a Thermo Scientific ESCALAB 250XI photoelectron spectrometer.

### Electrochemical Measurements

For the electrochemical measurements, conventional CR2032 type coin cells with the Fe_2_O_3_ nanocrystalline film anodes were assembled inside an argon-filled glove box with the oxygen and moisture content below 0.1 ppm. The electrochemical cells were prepared using lithium metal as the counter electrode and a standard electrolyte of 1:1:1 ethylene carbonate (EC)/dimethyl carbonate (DMC)/LiPF_6_. Galvanostatic cycling measurements were processed at room temperature by a LAND-CT2001A battery system at various current rates between 0.01 and 3.0 V. Cyclic voltammetry (CV) and AC impedance measurements were performed with a CHI660E electrochemical workstation (CHI Instrument TN). The scanning rate was 0.1 mV s^− 1^.

## Results and Discussion

**Fig. 1:**
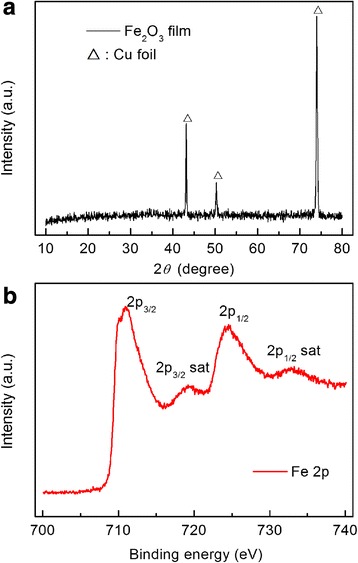
Structure and composition characterization of Fe_2_O_3_ film deposited at room temperature. **a** XRD patterns of Fe_2_O_3_ film. **b** XPS spectrum of Fe_2_O_3_ film

**Fig. 2:**
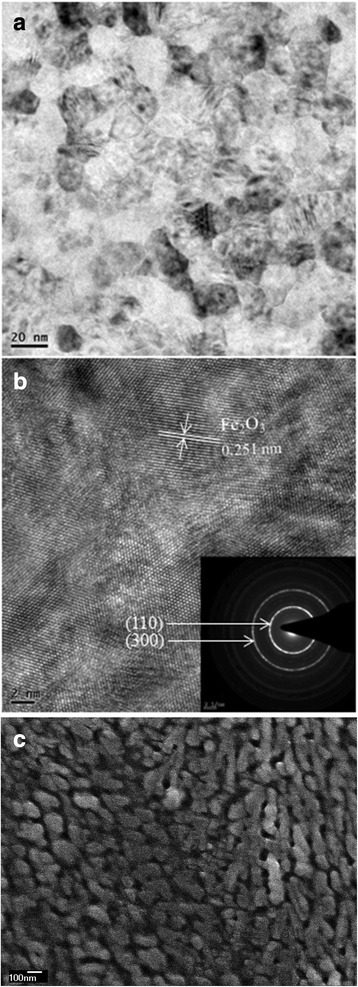
**a** TEM image. **b** HRTEM image with inset showing SAED patterns. **c** SEM image of the Fe_2_O_3_ film prepared at room temperature

X-ray diffraction (XRD) patterns of the Fe_2_O_3_ film are shown in Fig. 1a. It can be observed that there is no obvious peak except the peaks of cubic crystal Cu substrate, suggesting that the Fe_2_O_3_ film is amorphous or crystallized with nanosized grains. Such phenomenon could be attributed to the deposition occurred at room temperature. In order to determine the chemical composition of the obtained film, XPS measurement was performed as shown in Fig. 1b. The Fe 2p_3/2_ and Fe 2p_1/2_ main peaks are clearly accompanied by satellite structures on their high binding-energy side, with a relative shift of about 8 eV. The peaks of Fe 2p_3/2_ locating at 710.9 eV and Fe 2p_1/2_ locating at 724.5 eV are similar with XPS spectra of Fe_2_O_3_ reported in the literature [37–39]. To further reveal the structure and composition of as-deposited thin films, TEM characterization was conducted as shown in Fig. 2. It revealed that the Fe_2_O_3_ films were made of small nanograins with average size of several tens of nanometers. The HRTEM image clearly presents the lattice fringes of the (110) corresponding to d-spacing of 0.251 nm of α-Fe_2_O_3_. Meanwhile, the ring-like feature of the selected area electron diffraction (SAED) confirmed the polycrystalline nature of Fe_2_O_3_ film. As shown by the SEM images in Fig. 2c, the Fe_2_O_3_ film consists of particles in nanometer scale. Based on all these results, we can confirm that the film deposited at room temperature is composed of Fe_2_O_3_ with ultrafine nanosized crystalline grains.

**Fig. 3:**
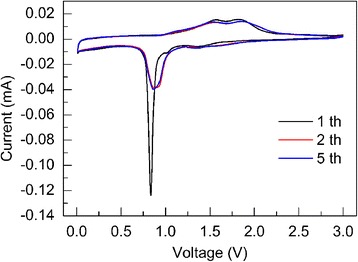
Cyclic voltammetry curves of the nanocrystalline Fe_2_O_3_ film. The curves were measured at a scan rate of 0.1 mV s^− 1^ from 0.01 to 3 V

The electrochemical performance of the electrode made of Fe_2_O_3_ nanocrystalline film was firstly evaluated by cyclic voltammetry (CV). Figure 3 shows the first three CV curves of Fe_2_O_3_ nanocrystalline film anode. The CV curves are similar to the previous reports of Fe_2_O_3_ anode [40–46]. In the first cathodic process, three peaks were observed at 1.38, 1.02, and 0.84 V, which could be related to a multi-step reaction. First, the very small peak at 1.38 V may be due to the lithium insertion into the crystal structure of Fe_2_O_3_ film forming Li_x_Fe_2_O_3_ without change in the structure [40, 43]. Second, another peak at about 1.02 V could be ascribed to phase transition from hexagonal Li_x_Fe_2_O_3_ to cubic LiFe_2_O_3_. The third sharp reduction peak at 0.84 V corresponds to the complete reduction of iron from Fe^2+^ to Fe^0^ and the formation of solid electrolyte interface (SEI). In the anodic process, two broad peaks observed at 1.57 and 1.85 V represent the oxidation of Fe^0^ to Fe^2+^ and further oxidization to Fe^3+^. In the subsequent cycles, the reduction peaks were replaced by two peaks locating around 0.88 V because of the irreversible phase transformation in the first cycle. The overlapping of the CV curves during the following 2 cycles demonstrated good reversibility of the electrochemical reactions, and this was further confirmed by the cycling performance.

**Fig. 4:**
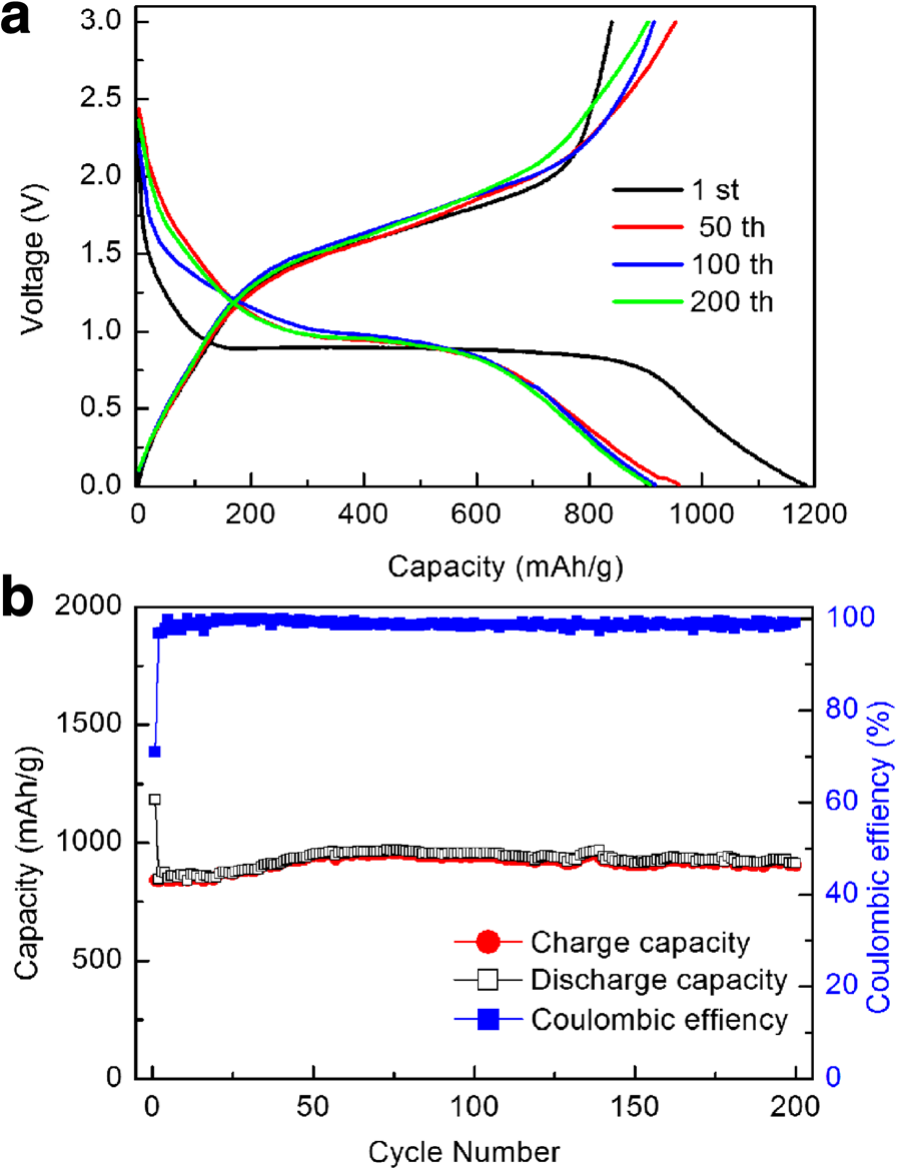
**a** Discharge-charge profiles of the nanocrystalline Fe_2_O_3_ film anode cycled between 0.01–3 V at a specific current of 100 mA g^− 1^. **b** Cycling performance of the nanocrystalline Fe_2_O_3_ film anode and corresponding Coulombic efficiencies at a specific current of 100 mA g^− 1^

Figure 4a shows the discharge and charge profiles of the Fe_2_O_3_ nanocrystalline film for different cycles at a specific current of 100 mA g^− 1^ with a voltage range of 0.01–3 V. Obvious voltage hysteresis are observed due to the conversion reaction during charge/discharge processes, and the voltage plateaus are in good agreement with the above CV results. The clear voltage slopes observed in each charge/discharge process indicate the oxidation of Fe to Fe^3+^ and the reduction of Fe^3+^ to Fe, respectively. The smooth slope from 1.5 to 2.0 V in the charge process represents the two oxidation peaks in the CV curves. Meanwhile, the plateau or slope around 0.9 V in the discharge process represents the reduction peak in the CV curves. The initial discharge and discharge capacity of the Fe_2_O_3_ nanocrystalline film are 1183 and 840 mAh g^− 1^, respectively, resulting in a Coulombic efficiency of 71%. The irreversible capacity loss is mainly attributed to the formation of SEI layer on the surface of anode, which is commonly observed in most anode materials [44–47].

The cycling performance of the film electrode at a specific current of 100 mA g^− 1^ at room temperature is shown in Fig. 4b. It can be seen that the reversible capacity gradually increases to 951 mAh g^− 1^ after the 70 cycles and then keeps stable in the range of 900–950 mAh g^− 1^ with a Coulombic efficiency nearly 100% during the following cycles. Similar phenomenon of the capacity increasing during cycling has been found in many transition metal oxide electrodes in previous studies [13, 48–52]. The possible reason for this would be the electrode activation, which induces the reversible growth of polymer/gel-like films to increase the capacity at low potentials [50]. Compared with the previous reports of Fe_2_O_3_ film anode batteries deposited by pulsed laser deposition or sputtering [35, 36], the capacity of Fe_2_O_3_ in our work has a considerable improvement as summarized in Table 1.

**Table 1:** The capacity comparison of our work with reported Fe_2_O_3_ film anode

Fe_2_O_3_-based thin film anode	Current density	Cycle number	Capacity (mAh g^−1^)	Ref.
Pulsed laser deposition	100 mA g^−1^ 100 mA g^− 1^	200 50	905 361	This work
Pulsed laser deposition	100 μA cm^− 2^	50	280	[35]
Sputter deposition	165 mA g^−1^	120	330	[36]

**Fig. 5:**
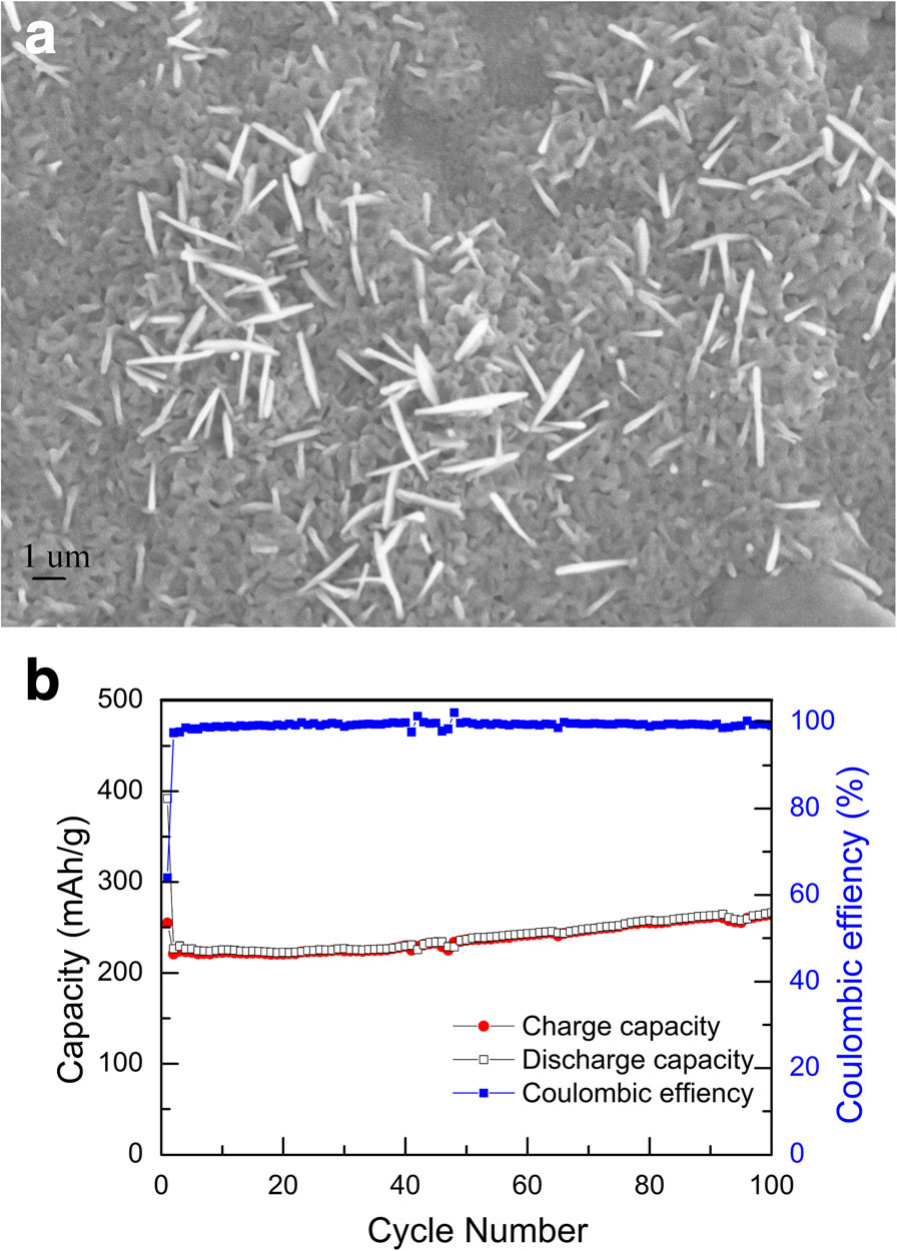
**a** SEM image and **b** cycling performance of the Fe_2_O_3_ film anode annealed at 400 °C at a specific current of 100 mA g^− 1^

**Fig. 6:**
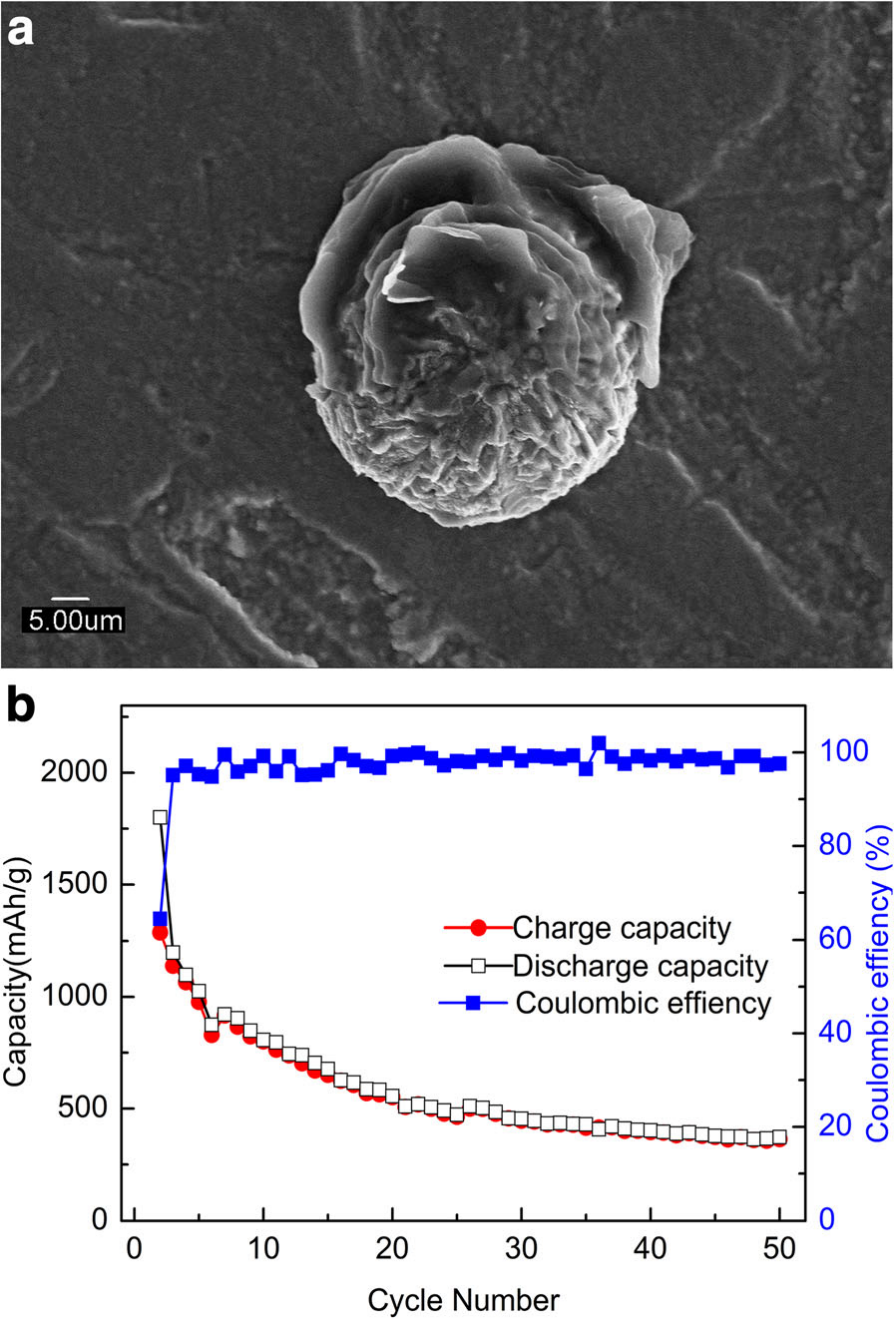
**a** SEM image and **b** cycling performance of the Fe_2_O_3_ film anode grown at 400 °C at a specific current of 100 mA g^− 1^

Previous studies on the effect of particle size on lithium intercalation into Fe_2_O_3_ shows that nanocrystalline Fe_2_O_3_ exhibited better electrochemical performance than macro-sized (> 100 nm) Fe_2_O_3_ [53]. To confirm the role of particle size in the electrochemical performance, we annealed the as-prepared Fe_2_O_3_ film on stainless steels at 400°. The prepared Fe_2_O_3_ film anode at high temperature was deposited on stainless steels only due to the instability of copper foil. The morphology comparison in Fig. 5a and Fig. 2c confirms that the particle sizes of the samples annealed at high temperature are obviously larger. Figure 5b shows that the capacities was only about 263 mAh g^− 1^ after 100 circles, which was much lower than the specific capacity of as-prepared Fe_2_O_3._ In addition, we also fabricated Fe_2_O_3_ film anode with larger particle size on stainless steels under 400 °C as shown in Fig. 6a. Figure 6b shows its discharge and charge profiles for different cycles at a specific current of 100 mA g^− 1^. The capacities dropped to 361 mAh g^− 1^ after 50 circles. These results indicate that the enhanced reversible capacity of nanocrystalline Fe_2_O_3_ film grown at room temperature can be attributed to the nanoscaled structure of the thin film electrode, which can sustain high lithium insertion strain because of the smaller number of atoms and large surface areas within nanoparticles [13, 14, 54].

**Fig. 7:**
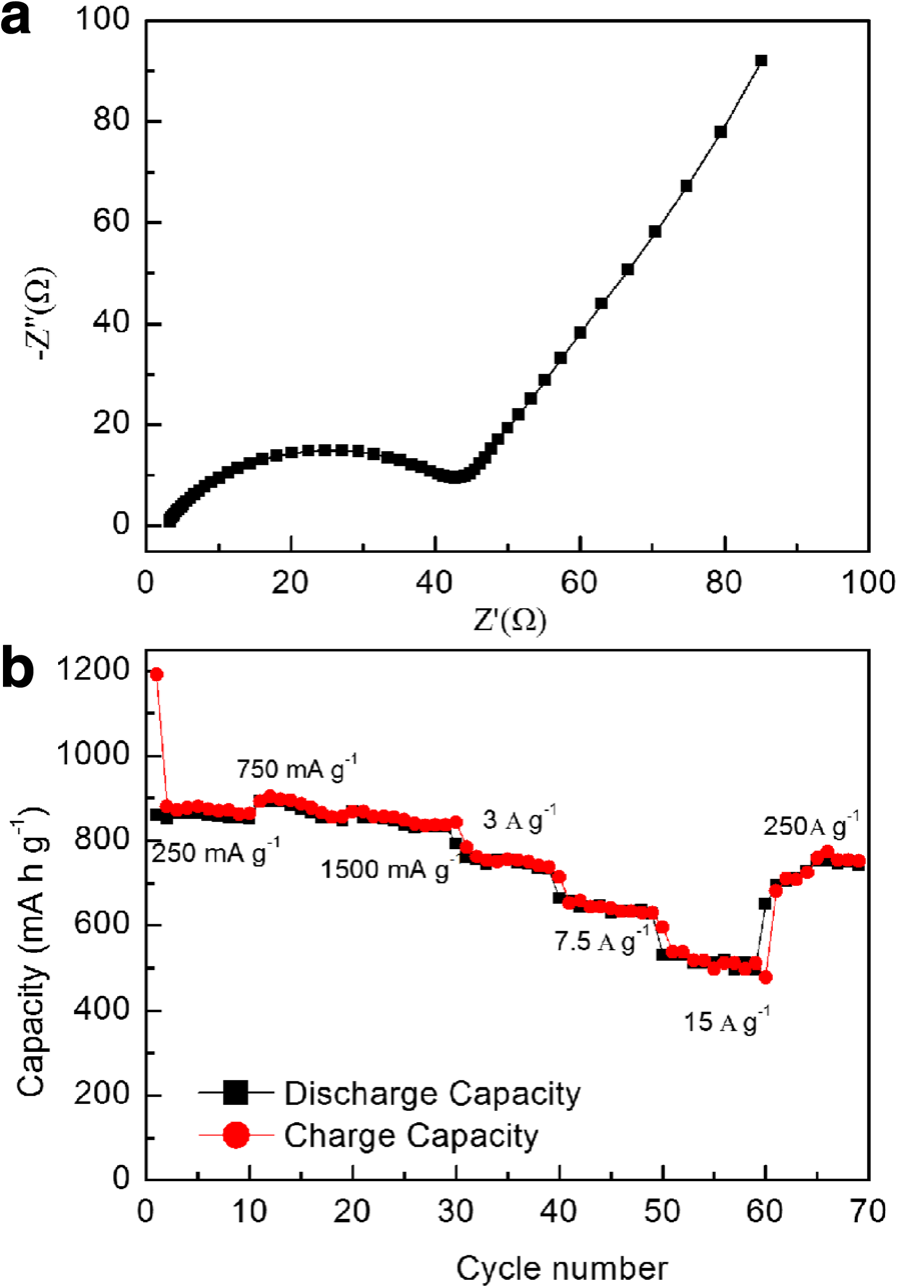
**a** Electrochemical impedance spectra of the nanocrystalline Fe_2_O_3_ film. **b** Rate capabilities of the nanocrystalline Fe_2_O_3_ film at different specific currents

To investigate the kinetics of lithium inserting/deinserting, electrochemical impedance spectra measurement was performed in Fig. 7a. The charge-transfer impedance on the electrode/electrolyte surface is about 50 Ω, which can be deduced from the single semicircle in the high-middle frequency. The superior conductivity of the film electrode without binder can be attributed to the nanocrystalline structure of the Fe_2_O_3_ film and the enhanced electrical contact between active anode and substrate. The good conductivity of the nanocrystalline Fe_2_O_3_ film anode led to excellent rate performance. Figure 7b shows the charge/discharge capacities at different current densities. The anode delivered capacities up to 855, 843_,_ 753, 646, and 510 mAh g^− 1^ at high current densities of 750, 1500, 3000, 7500, and 15,000 mA g^− 1^, respectively, which is corresponding to 98.2, 96.7, 87.8, 75.3, and 59.5% retention of the capacity at 250 mA g^− 1^ (about 871 mAh g^− 1^). More importantly, when the specific current reduced to 250 mA g^− 1^, the capacity could recover to 753 mAh g^− 1^. The excellent rate performance benefits from both the good conductivity of the anode and the increase of capacity upon cycling.

## Conclusions

In summary, nanocrystalline Fe_2_O_3_ film anode has been deposited by pulsed laser deposition at room temperature. The results of structure and morphology characterization showed that the deposited films are composed of nanocrystalline Fe_2_O_3_ with grain size of several tens of nanometers. The prepared Fe_2_O_3_ exhibits an excellent electrochemical performance, such as superior cycling stability (905 mAh g^− 1^ at a specific current of 100 mA g^− 1^ after 200 cycles) and high rate capability (510 mAh g^− 1^ at 15000 mA g^− 1^). The outstanding electrochemical performance can be related to the nanocrystalline structure of Fe_2_O_3_ which could sustain high strain, shorten diffusion lengths for lithium-ion, and keep the structure stable. The excellent electrochemical performance and room temperature growth suggest that nanocrystalline Fe_2_O_3_ has potential application in high performance LIBs, especially in all-solid-state thin film batteries.

## Abbreviations

AFM: Atomic force microscope
CV: Cyclic voltammetry
DMC: Dimethyl carbonate
EC: Ethylene carbonate
LIB: Lithium-ion batteries
PLD: Pulsed laser deposition
SEAD: Selected area electron diffraction
SEI: Solid electrolyte interface
SEM: Scanning electron microscopy
TEM: Transmission electron microscopy
TMOs: Transition metal oxides
XPS: X-ray photoelectron spectroscopy
XRD: X-ray diffraction
